# Potential roles of pharmacists in HIV/AIDS care delivery in Nepal: A qualitative study

**DOI:** 10.1371/journal.pone.0280160

**Published:** 2023-01-06

**Authors:** Ayushma Shahi, Sweta Shrestha, Badri K. C, Khagendra Acharya, Sait Kumar Pradhan

**Affiliations:** 1 Department of Pharmacy, School of Science, Kathmandu University, Dhulikhel, Kavre, Nepal; 2 Department of Management Informatics and Communication, School of Management, Kathmandu University, Dhulikhel, Kavre, Nepal; 3 Department of Clinical Physiology, Maharajgunj Medical Campus, Institute of Medicine, Kathmandu, Nepal; Torrens University Australia, AUSTRALIA

## Abstract

**Background:**

Nepal is facing escalating infection rates of HIV/AIDS, a major global public health threat. Continuum of services is an identified strategic component of Joint United Nations Programme on HIV/AIDS (UNAIDS) commitment to end this public health crisis by 2030 and achieve the Sustainable Development Goal 6 (SDG 6). Pharmacists are integral members of the continuum of care in HIV/AIDS but the idea is novel to Nepal. Realizing need to explore and identify potential roles of pharmacists in HIV/AIDS care delivery, this study aimed to gain an insight into the views of stakeholders on the roles of pharmacists in this arena.

**Methods:**

A qualitative approach was used where 14 key informants were interviewed using a semi-structured interview protocol. Participants were selected through a sequence of purposive sampling and snowball sampling technique. The interviews were conducted, transcribed verbatim and analyzed using thematic analysis.

**Results:**

Potential roles of pharmacists reside in adherence monitoring, pharmacovigilance, provincial and district level ART centers. Pharmacists and other stakeholders held divergent views on the pharmacist’s role in dispensing and counseling antiretroviral medications. Barriers to the pharmacists’ involvement were lack of workforce, advocacy and government support, frailty of professional organizations, self-limited scope, policy constraints, structural limitations, biasedness, and societal unawareness. Pharmacists themselves and organizations such as National Government Organizations (NGOs) and International Government Organizations (INGOs) were identified as the facilitators.

**Conclusion:**

Stakeholders are willing to expand role of pharmacists in HIV/AIDS care in Nepal. Nevertheless, some crucial impediments exist. Primarily, an aggressive and assertive advocacy is needed from pharmacists themselves and their professional organizations to establish their roles in HIV/AIDS care delivery. Additionally, unearthing potential of pharmacists as contributors in HIV/AIDS care delivery or any other chronic disease management equally demands a strong support from the government officials as well as the other health care professionals.

## Introduction

HIV/AIDS continues to be the major global public health issue claiming 40.1 million lives as of year 2021 [1, 2]. As per WHO 2022 data, out of 38.4 million people living with HIV globally, 3.8 million reside in Southeast Asia [3]. After sub-Saharan Africa (25.6 million), South East Asia (3.8 million) has the second highest prevalence of HIV [4, 5]. Nepal, one of the countries in South Asia, is facing a rapid surge in infection rates since the detection of first HIV case in 1988, with the current estimation of PLHIV being 29,503 and the adult (15–49 years) HIV prevalence rate being 0.13% [6].

Since the detection, Nepal has put substantial efforts such as launching the first National AIDS prevention and control program in 1988 [7]. Furthermore, National Centre for AIDS and STD Control (NCASC) in 2008 provided an extensive training to hundred pharmacists on HIV testing and Antiretroviral (ARV) monitoring, a reflection of an effort to develop trained human resources for HIV/AIDS programme [8]. As an exemplar of the global endeavor, the International Pharmaceutical Federation (FIP), in collaboration with World Health Organization (WHO), called attention to important public health issues, including the role of pharmacists in fighting against the HIV-AIDS pandemic and encouraging adherence to long-term treatments [9].

The National Strategy on HIV/AIDS (2006–2011) had strongly emphasized the importance of a continuum of services from prevention to treatment and care where pharmacists play a crucial role. Internationally, an ambitious treatment plan aiming to end the AIDS epidemics by 2030 is being implemented whereby all Health Care Providers involved in HIV care have their clear and defined roles in the service [10]. Along the same strategy, many countries view pharmacists as critical members of the continuum of care and numerous articles highlight the positive impact of pharmacist-provided HIV/AIDS care [11, 12]; in Nepal, however, these arenas remain obscure. In the light of this scenario, it is noteworthy to document the current and potential future role of pharmacists as service providers in Nepal’s overall management of HIV/AIDS. Realizing the lacuna, this study is initiated to uncover the potential role of pharmacists in HIV/AIDS care delivery in Nepal.

Pharmacists are recognized as one of the essential and integral members of the HIV care team widely across countries like the USA and Canada [13]. There was a time when HIV/AIDS was considered incurable, and patients were left without any chances of survival; it was during such crucial time pharmacist had played their role. Advent of ART has successfully transfigured HIV/AIDS into a chronic manageable disease. With these advances, the goal of treatment is also constantly shifting. While the primary goals of HIV therapy remain maximal viral suppression and immune reconstitution via combination ART, secondary goals include long-term adherence, avoiding drug interactions, minimizing toxic effects, simplifying treatment regimens, lowering drug costs, managing co-morbid conditions and preventing HIV transmission [14]. As reported in available studies [12, 15, 16], when community pharmacists provide HIV-focused services that go beyond the contemporary practices, a shift occurs. Pharmacists’ involvement in HIV care delivery is associated with increased adherence [17], reduced dosing frequency and pill burden, improving outcomes [18], decreased CD4+ cell count, viral load, and drug-related toxicities [19]. In countries like the United States of America and Canada, pharmacists’ roles in HIV care have been observed in many of these domain [13]. Furthermore, to address new challenges, these countries are constantly identifying and establishing new roles in care [11]. While the pharmacist’s role in HIV/AIDS care is well established in these countries, it has received insufficient attention in Nepal. The pharmacy profession in Nepal is still in its infancy, hence most pharmacists are involved in community or hospital pharmacies, academics, or the pharmaceutical industry. It is high time to explore, identify and establish new roles with emerging challenges in HIV care delivery, acknowledging the absolute lack of such studies in Nepal. Hence, this study is an attempt to ascertain pharmacists’ current and potential involvement in HIV/AIDS care delivery in Nepal.

## Methods

### Study design and study site

This was a qualitative study where we diverged into the exploratory aspects of pharmacists’ involvement in HIV care as there was no prior findings to rely on. Thus, to explore stakeholders’ understanding about pharmacist’s current role, potential roles, barriers and facilitators, we designed a qualitative study. The study sites included the organizations that are working for HIV/AIDS patients. Major organizations include: the office of National Centre for AIDS and STD Control (NCASC), Department of Drug Administration (DDA), Nepal Pharmacist Association (NPA), Nepal Pharmacy Council (NPC), Save the Children, Ministry of Health and Population (MOHP), and Logistic Management Division (LMD).

### Ethical approval

Ethical approval (NHRC- Registration no. 834/2020 P) was obtained from Nepal Health Research Council (NHRC) before the data collection. Following the approval from NHRC, the study objectives were explained to the respondents, and written informed consent was obtained from each participant before the interview. In addition, permission was taken from the respondents to record the interview with an assurance of maintenance of confidentiality.

### Study participants and their recruitment

Most of the study participants were the individuals working in the organizations related to HIV/AIDS. One of the authors (BKC) was involved in HIV/AIDS care sector in his earlier phase of career. Through him some key informants from the policy making level and from service provider level were identified initially. The official website of National centre for AIDS and STD control (NSASC) was also utilized to identify initial key informants, such as pharmacists involved in HIV/AIDS care delivery. Similarly, policymakers including the director of NCASC, the secretary of the Ministry of Health and Population, the director of the Department of Drug Administration, the Nepal Pharmacy Council, and the Nepal Pharmaceutical Association were identified via their respective institutional websites. On this basis, an initial list of key informants was compiled.

The second phase involved contacting the key informants via Facebook or in-person after scheduling an appointment. On Facebook, two NCASC-employed pharmacist informants were located and sent a message pertinent to the study’s objectives. Following their affirmative response, a formal appointment with the director of NCASC was scheduled. The purpose of the study was thoroughly explained to the director during the meeting, and a formal letter requesting the collection of data was submitted. After approval, appointments were requested from pharmacists and the NCASC director. Similarly, other policymakers were approached through formal appointment and the same procedure was followed. A total of 14 participants were interviewed. Two predetermined key informants did not participate because they deemed another informant to be more credible and referred them. The other informants were selected following snowballing technique. The respondents for this study are mentioned in Table 1.

**Table 1:** Participants of the study.

No.	Participant	Number	Institution
1.	Medical doctor	1	Director, NCASC
2.	Pharmacist	1	Former Director, DDA
3	Pharmacist	2	NCASC, logistic department
4	Pharmacist	2	Save the Children
5	Pharmacist	1	Former employee, Save the Children, FHI360
6	Pharmacist	1	LMD
7	Pharmacist	1	Registrar, NPC
8	Pharmacist	1	MOHP
9	Pharmacist	1	Secretary, NPA
10	Public Health Officer	1	LMD
11	Public Health Officer	2	NCASC

The appointments for interview were taken according to the informant’s convenience. Time and place for interviews were selected by the key informants. All the interviews were conducted face-to-face, and only interviewer and interviewee were present during interviews. At the end of each interview, the informants were asked for any potential informant. The contact number of the potential informants were sought, and they were contacted through telephone call. Then, appointment of the interview was taken. Four interviews were done after office hours in the cafeteria, one in the office canteen, two virtually via zoom, and seven during formal office hour in the key informant’s respective office.

### Data collection

Interview guides were used to conduct the interviews. Three interview guides were developed both in English and Nepali: one for pharmacists (Appendix 1 in S1 File), one for policymakers (Appendix 2 in S1 File) and one for other healthcare professionals (Appendix 3 in S1 File). The interview guides were designed after an extensive literature review and series of discussions within the team. The interview questions focused on pharmacists’ perceived roles in HIV/AIDS care, their impact, the need for pharmacists, and perceived facilitators and barriers to pharmacist’s involvement in HIV/AIDS care.

A series of practice sessions were run prior to final data collection where the interviews were conducted with the supervisors to ensure proper delivery, intelligibility of the questions, and further refinement of the interview guide. After finalizing the interview guide, it was subjected to pilot testing where two interviews were conducted with relevant stakeholders. Necessary modification was done in the interview guide following the pilot testing. For instance, the question “There seems to be no role of pharmacist in Antiretroviral (ARV) related dispensing, counselling and adherence monitoring in Nepal, why do you think so?” was split to two questions “In the context of Nepal, who is involved in dispensing, counselling and adherence monitoring of ARVs?” and “In your opinion, is it necessary to involve Pharmacist in activities like dispensing, counselling, adherence monitoring related to ARVs?” The two pilot interviews were excluded from the final data analysis. Data was collected from March 2021 to June 2021. AS conducted all fourteen interviews. All the interviews were audio-recorded, and field notes were taken during the interview. The interviews ranged from 30 minutes to one hour. No further interviews were conducted after data saturation. When AS began to hear similar responses, data saturation was believed to have been reached, indicating that it was time to stop collecting data and begin analysing what had already been gathered [20].

### Data analysis

To analyze the data, Braun and Clarke’s six step process—familiarizing with the data, generating initial codes, searching for themes, reviewing themes, defining and naming themes, and producing the report—was used [21]. To familiarize with the data, the audio-recorded interviews were transcribed verbatim by AS and all the transcripts were circulated among the team with the request to read thoroughly. After thorough readings, two authors (AS and KA) were assigned to do coding, and it was agreed that translation and back translation of the interviews conducted in Nepali language would be done after coding. Following these decisions, AS and KA independently coded the data, and all coded extracts from the interviews in Nepali language were translated into English by AS; and to ensure quality standard, some randomly selected extracts from the translated version were back-translated into Nepali by KA. Since very few and minor differences were observed in the two versions, English-translated version was considered appropriate. Initially, the coding process was done inductively, and after coding seven transcripts, all authors discussed the codes for emerging themes. Any discrepancies in the codes derived by AS and KA were settled unanimously, and the cluster of codes were prepared to figure out emerging themes. The emerging themes were clustered into Current role, Disagreement among stakeholders, Potential role, Barriers and Facilitators. On the basis of these themes, a framework was developed, and the remaining seven transcripts were coded. In the third stage, all the codes clustered into different categories were sorted to identify potential themes and subthemes. While reviewing and refining themes and subthemes all the compiled excerpts were re-read to determine if they appeared to form a logical pattern. A thematic map was also prepared to ensure that the themes and subthemes cohere, and they reflect meaning of the excerpts. Next to this stage, the themes were named on the basis of the value they captured. Then, they were defined. In the final stage, a report based on the themes and subthemes was prepared [22, 23].

## Results

The analysis of interview excerpts revealed five themes related to the topic of this study, the potential roles of pharmacists in HIV/AIDS care delivery in Nepal. The themes include Current roles, Disagreement among stakeholders, Potential roles, Barriers, and Facilitators. All these themes along with the excerpts are presented below:

### Current roles

The theme—current roles—highlights the role of pharmacists in HIV/AIDS care provision in Nepal, and also entails the existing status of pharmacists in Nepal. Our findings indicate that pharmacists are currently involved only in central logistic management. All respondents directly involved in HIV/AIDS care reported that two central offices, NCASC and Save the Children, are responsible for the procurement and distribution of ARVs to various ART centers in Nepal. Pharmacists only participate in logistic management in these central offices. The respondents directly involved in care, both pharmacist and non-pharmacists identified the similar roles.

The following excerpts summarize the roles of pharmacists. One pharmacist stated “*one of my roles is procurement*, *storage and supply chain management “#KI03*. Another said *“during procurement*, *we look over quality assurance and technical evaluation their main role is procurement and supply management” #KI02*. Yet, the other revealed, *“Our role or our job is not directly clinical*, *but is the procurement of HIV-related drugs*, *their successful storage and distribution” KI05*. Summing up, it can be concluded that pharmacists are confined to non-clinical engagements.

Non-pharmacist respondents’ statements also substantiated what pharmacists stated. One said, “*their main role is procurement*, *warehousing and supply management” #KI08*. And, another respondent remarked, *“After procurement*, *we ask the pharmacists to do the quality assurance” #KI09*.

Only pharmacist respondents identified providing training on logistic management as their current roles whereas both pharmacist and non-pharmacist respondents highlighted providing support to the ART centers. As articulated by a pharmacist “*We provide support to ART centers…*. *they reach out to us regarding the pediatric dose*, *so we calculate the dose…*.*” #KI04*. Another stated, *“Sometimes*, *we calculate pediatric dose" #KI03*. Here, two pharmacists specified supportive role as pediatric dose calculation, while non-pharmacist respondent just acknowledged their support to the ART centers vaguely, “*Problems keep coming from the district*. *Even though we all tackle the problem together*, *they are the main ones*. *They are the first contact*.*” #KI12*. The pronoun used here, “they,” refers to the pharmacists. The pharmacists being the “main ones” and “first contact” acknowledge the role of pharmacists but does not accredit the pharmacists exclusively.

To sum up, this theme shows the current roles of pharmacist in HIV/AIDS care delivery in Nepal is Logistics management, which involves procuring ARVs, ensuring their quality, warehousing them, managing the supply chain, ensuring a smooth flow of logistics, providing training, and support to the ART centers.

Additionally, despite the fact that the majority of informants (80%) were aware of the current number of pharmacists involved in HIV care, even the responsible pharmacists were unsure of the number. For example, one pharmacist said, “*There are… wait*… *1… 2 pharmacists” #KI03*; and another stated, *“there are 1*, *2*, *3…*.*5 pharmacists now” #KI04*. The responses, “There are… wait… 1… 2 pharmacists,” and “there are 1, 2, 3…5” may be revealing uncertainty of the pharmacists about the fellow members working in the same field. What they apparently speak is the existence of a very small number of pharmacists, and the insignificance given to pharmacists.

### Disagreements among stakeholders

The theme- disagreement among stakeholders- highlights the existing dispensing and counselling practices on ARVs, and key informants’ opinions on the same.

Only the respondents directly involved in HIV/AIDS care were aware of the current ARV related dispensing and counselling practices. Dispensing and counselling were reportedly carried out by trained ART counsellors with nursing or public health background, or Health Assistants (HA) and Auxiliary Nurse Midwife (ANM). The respondents clearly stated the roles which is mentioned in the excerpts below:

- *KI06*: *Dispensing and counselling are done by HA*, *the staff nurse*. *They are trained*.
- *KI01*: *PCL nursing dispenses*. *At some centers*, *there is Bsc*. *Nursing*, *BN*, *or those with public health degree…and in some places*, *there is HA*. *However*, *maximum there are either HA or staff nurse*.

Pharmacist respondents’ statements presented above strongly point to the reality that they were the potential untapped resources for undertaking this responsibility.

There was a general disagreement among pharmacists and non-pharmacists respondents regarding the role of ARV related dispensing and counselling. Pharmacists were of the opinion that their involvement in ARV dispensing and counseling would create a positive impact in terms of increased adherence and better facilities. Pharmacists were adamant that the roles of dispensing and counseling are theirs, citing their respective expertise to support their claim. One pharmacist suggested “Others *are not allowed to dispense or counsel*. *It is illogical…*. *there should be a pharmacist…*. *pharmacists have in-depth knowledge*, *and they can counsel patients accordingly” #KI14*. And, another pharmacist stated “…*pharmacist*, *we mean medicine expert*… *dispensing and counseling is the role of the pharmacist” #KI14*. Both these assertions place pharmacists as the major responsible persons for ARV dispensing and counseling. Pharmacists also emphasized that their involvement in ARV dispensing and counseling would create a positive impact in terms of increased adherence and better facilities. One pharmacist said “*pharmacist can deliver far more and better facilities to the patients” #KI01*.

While majority of pharmacists revealed strong opinions of pharmacists regarding their potentially beneficial roles; some, however, were ambivalent. Respondents identified dispensing and counseling as pharmacist roles but did not deny that any trained individual could perform those duties as well. One pharmacist stated “*if a specified person is well-trained*, *then we cannot argue that the person cannot perform well*, *but certainly*, *pharmacists are well-placed” #KI11*

Non-pharmacist respondents in contrary to the pharmacist respondents held a contrary view. The respondents in public health felt that dispensing and counseling on ARV is not pharmacist’s role. “Absence of roles” “Absence of visible roles” and “Absence of a need for pharmacists in dispensing and counselling” were concurrent codes in their statements. One respondent said “*This role is different*. *This role belongs to monitoring*, *evaluation*, *treatment*, *and care*, *and I do not think this is pharmacists’ role*.*” #KI09* The outright denial, which is a representative quote, can be considered as a manifesto of non-pharmacists’ take on the issue of dispensing and counseling on ARV.

Pharmacists were deemed superfluous in dispensing and counselling ARVs. Staff nurses and health assistants were the ones who took ownership of this role. One respondent asserted, “*there are counselors plus staff nurses or those with a medical background …*. *I do not think pharmacists counsel the patient*, *most probably*, *counseling can be done only by the trained counselors*.*” #KI08*. This excerpt demonstrates that the informant does not consider pharmacists to be medical professionals. It is implied that only those with a medical background are qualified to dispense and counsel, and pharmacists are ineligible.

Additionally, budgeting was cited another reason for not hiring a pharmacist in dispensing and counselling. One stakeholder quoted “*There are 80 ART centers*, *and we have not been able to retain even doctors in all of them because of budgeting issue*, *so*, *lets’ not speak about others*. *So*, *one there is budgeting*, *and another there is no role visible role of the pharmacists*.*” #KI12* Here, the informant describes how budgeting has been an issue to retain professionals. The informant in the excerpt KI12 emphasized especially doctors quoting "even doctors" which clearly can be assumed that only doctors are considered important.

### Potential roles

The theme—potential roles—spotlight the informants’ perspective on the roles that pharmacists can play in HIV/AIDS care delivery. The theme has two subthemes, a) logistics management, and b) pharmacovigilance and adherence monitoring.

#### Logistics management

While our results show the current roles of pharmacist at central level logistic management, majority of participants emphasized the importance of pharmacists in logistical management at other levels, such as district, provincial, and regional ART centers. They cited the efficiencies of pharmacists and their expertise in logistics management as crucial roles, and they were confident that their participation at other levels would be advantageous. For instance, one pharmacist said “*If pharmacists are involved*, *then there are many benefits like warehousing*, *storage*, *and QA” #KI020*. Similarly, one non-pharmacist respondent said “*Pharmacists are needed in the provincial sectors*. *That kind of knowledge will help them take measures to smoothen the supply chain”* #*KI08*. Both these statements point to the importance of involving pharmacists in logistic management.

#### Pharmacovigilance and adherence monitoring

Around 53% of respondents indicated that pharmacists could play a role in pharmacovigilance and adherence monitoring. Of the 53%, 50% used the term pharmacovigilance directly, while the remaining referred adverse drug reaction and adverse effect monitoring. Similarly, adherence monitoring was also highlighted to be one of the potential roles of the pharmacist. One pharmacist responded “*There is a huge role in pharmacovigilance” #KI05*. Another stated, *“*… .*it is necessary to manage the side effect of those medicines…*. *So*, *in that sector*, *like pharmacovigilance*, *QA…*. *if there is someone from a pharmacy background because they have that kind of knowledge and*… *they have worked in manufacturing so*, *they can support manage the program*.*" #KI03* The assertion in the first quote and its reiteration along with the rationale reveal that the pharmacists believe pharmacovigilance and adherence monitoring as their own responsibilities.

### Barriers

The theme- barriers- spotlight the hindrances to the pharmacists’ involvement in HIV/ AIDS care delivery in Nepal. It is further categorized into three subthemes, a) pharmacist-related weakness, b) government-related weakness, and c) society-related weakness.

#### Pharmacist-related weaknesses

The pharmacist’s perceived inability to advocate through authority figures such as pharmacy professional organizations and those involved in higher-level governmental bodies was cited as a reason for pharmacists’ lack of success in the field. Many important barriers related to this aspect were identified by the key informants. The most prominent code in this subtheme was lack of advocacy. Majority of the respondents pointed out the lack of advocacy from the professional organization, such as GPAN (Graduate Pharmacists’ Association Nepal), NPC (Nepal Pharmacy Council), and NPA (Nepal Pharmaceutical Association). The following excerpt represents the voice of many pharmacists: “*We have not been able to put forward our voices to the authority*. *I do not think our professional organizations like GPAN*, *NPA have been working on that level” #KI04*. Another respondent explained, *"the reason why pharmacists are not in ART centers is because of weaknesses in the policy level*, *meaning those among our professionals who are involved in policy level*, *it is because of their weaknesses” #KI03*. Similarly, policy makers pointed to this reason. One of them reasoned “*…*. *it is because of insufficient advocacy*. *Maybe you have your professional association or organizations*, *so you have to increase your self-value through them” #KI12*. The understanding of the policy maker echoed in another pharmacist’s explanation: *“We have pharmacist associations and different kinds of organizations*, *and these organizations have failed to advocate” #KI11*.

Secondly, pharmacists’ own shortcomings such as lack of awareness, interest, willingness and a self-contained scope of the practice was reported as a major barrier. One pharmacist said “*There is no such demotivation factor but only unwillingness among pharmacists that is holding them back” #KI03*. Similarly, self-limitation of the pharmacists in manufacturing company and hospital pharmacy was popularly expresses barrier by the respondents. For example, one respondent said, “*As soon as someone finishes their degree*, *they go straight to the manufacturing company” #KI05*. Similar ideas were expressed by another respondent, *“Pharmacists’ are more engaged in the industrial side*, *and few of them are going to the hospital*, *but the clinical part is untouched” #KI14*.

Another barrier identified from the data was pharmacists’ lack of initiative to explore their roles in important areas as research. One policymaker quoted “*there is no research base at all*. *So*, *they should explore their roles rather than only doing what they are asked” #KI12*. Yet, another pharmacist said “*They are not exploring their role…*. *we do not have a solid research base…*.*” #KI03*. In both these excerpts, the failure to initiate research is highlighted as a major barrier.

Finally, another barrier under this subtheme was the lack of pharmacists in Nepal. One pharmacist said “*The reason of pharmacist not flourishing in HIV is firstly the number*.*…” #KI06*. The explanation for this lack was presented by one policy maker, “*There was no pharmacist topic previously*, *so this is new*. *Maybe it is because there are no pharmacists*. *There are no pharmacists in our sector*, *but we need them…” #KI12*.

#### Government-related weaknesses

This subtheme relates to the government’s shortcomings as a barrier to pharmacists participating in HIV care. “No government allotment” was the most frequently used code in this section, with 93% of participants stating that pharmacists are not assigned to HIV-related services. Pharmacists highlighted the lack of government’s allotment of their position in government services as one of the challenges encountered during the service. Participants expressed their dissatisfaction with the lack of government allocation. The following two remark, representing the voice of many pharmacists, stated “*government does not appoint us*. *We are hired by Global Funds and Save the Children*, *but we work for the government” #KI01*. The ‘government’ referred here is federal level government of Nepal; the government is alleged for not recruiting the pharmacists. A very sad note was expressed by another respondent, *“there are posts but few*. *I do not know if it is appropriate to say this as a government officer*, *but as a youth pharmacist*, *I would say it is saddening” #KI15*.

All the pharmacist participants indicated the pervasiveness of bias and domination in healthcare field as possible weakness on part of government. The code of ‘biasedness’ often appeared along with the code of domination. The following are the representative responses of the pharmacists. First excerpt: “*the policymakers are a bit biased*. *Let’s say if I were in that authoritative position then*, *I would prioritize my profession only” #KI03*. Second excerpt: *“the healthcare system in Nepal seems to be somewhat biased like they consider doctors to be an important person and have been unable to integrate other sectors” #KI14*. Third excerpt: *“doctors have been dominating since the beginning” #KI05*. The target of pharmacists’ blame in these responses is apparently Nepal government and the governmental bodies. The following response from a pharmacist reiterates the view:

“*There are 2–3 factors for this*. *One*, *lack of awareness*. *Second*, *there is no such vision or focus from government or say policy level”*

#*KI05*.

The policymaker respondents and non-policy maker respondents had divergent views regarding governmental constraints. The non- policymaker respondents assessed the current policies to be outdated, biased and non-specific. For instance, one respondent said “*Dispensing*, *counselling is done by staff nurses and HA*. *They made this policy*, *I guess around ten years ago*, *and*, *at that time*, *they had no idea about the need for pharmacists*.*…” #KI01*. Yet another respondent claimed, *“there are no specific policies” #KI06*. Importantly, pharmacists believed that if their roles were specified in policies, it would minimize the biasedness. One pharmacist stated “*If roles are clearly defined*, *everyone’s space is made clear*, *and roles are assigned per expertise in the existing health structure of the nation*, *then the biasedness will gradually minimize*. *“#KI14*

On the contrary, the policymaker respondents were of the view the organizational structure rather than policy was responsible for the biases. One policymaker cited “*I have heard that there is the provision for pharmacists’ involvement in the policy*, *but that has not been reflected in the organogram” #KI12*. This response demonstrates the policymakers’ uncertainty regarding the policy. However, this response is backed by another policymaker who stated “*There is a lack of structural requirements*. *For example*, *there should be a position for pharmacists where patients are treated*, *but the allotment arrangement could not be made*, *which is why there is an insufficient workforce leading to pharmacists being unable to provide services*. *So*, *a policy is not restricting*, *but the structure is creating problems” #KI11*.

Here, the structural constraints are pointed out to be the major problem creators, not the policies. The respondent further elaborated as “*Structure is made by the health ministry and Nepal government altogether*. *Health ministry has to propose*. *The related body determines the structure from a general health post to the tertiary care hospital and different departments*. *In this case*, *health ministry should propose*, *and that is lacking” #KI11*

#### Society-related weaknesses

This subtheme is concerned with pharmacists’ social recognition. One pharmacist said “*In the context of Nepal*, *we have not got social recognition yet*. *“#KI15* Societal unawareness and lack of social recognition were two prominent codes associated with this theme. Social oblivion was identified as one of the pharmacists’ challenges. The following representative quote elucidates the theme, “*our society or community are still unaware of the arrays of services we are capable of providing*. *They follow doctors and what you see now is the reflection of the whole society” #KI06*.

### Facilitators

The theme- facilitators- spotlight the perceived factors that facilitate the involvement of pharmacists’ in HIV/AIDS care delivery in Nepal. This theme contained two subthemes, a) pharmacist and b) organization.

#### Pharmacist

The first subtheme foregrounds pharmacists’ perspectives on self-satisfaction and recognition as a factor motivating or encouraging their involvement in PLHIV services. Pharmacists expressed an optimistic view of their capabilities and contribution. One pharmacist said “: *I feel happy to see the patients get the medicine on time” #KI01* The causal relation of pharmacist’s happiness and the patient receiving medicine on time clearly speaks to the fact that pharmacists’ find themselves satisfied even at small incidents. It was also articulated that their work in the field resulted in recognition of their significance. Another pharmacist stated “*As I started working*, *people began to realize my roles and importance… we have given results which is why nowadays they have started hiring pharmacists*.*” #KI05*. Both these statements, which are representative samples, imply that pharmacists consider their self-esteem as a facilitator.

#### Organizations

Participants expressed a favorable attitude toward the organizations working for HIV/AIDS patients. Global Fund and Save the Children were considered pioneers in involving pharmacists in HIV/AIDS-related services. Pharmacists cited that these organizations contributed positively to their increased involvement in care.

The following excerpts, for example, emphasize the role of non-governmental organizations: “*Global fund operates throughout the world*, *and since its operation in Nepal*, *people have come to know about the pharmacist especially their need in drug sector and distribution sector*. *This has led to many organizations hiring pharmacists’ especially in drug distribution and procurement*.*” #KI05*. A number of outcomes such as people knowing about the pharmacists, and organizations hiring pharmacists are attributed to non-governmental organizations. Similar ideas were expressed by another pharmacist: “*In other countries*, *pharmacists are widely involved in HIV*, *which is why Global Fund hired pharmacists*… . *they brought that concept in Nepal…*. *then everyone realized the need of pharmacist*.*” #KI02*. As in the previous excerpt, the role of Global Fund is acknowledged forthrightly.

## Discussion

This study examined the status of pharmacists in HIV/AIDS care delivery in Nepal as perceived by the stakeholders (pharmacists, public health officers, and policymakers). In the context of HIV/AIDS, no earlier study in Nepali context comparable to the present one was found. The data obtained from stakeholders addressed our research question regarding pharmacists’ current and potential roles in HIV/AIDS care and the barriers and facilitators to pharmacists’ involvement in the same. As of September 2021, there are 4945 registered pharmacists and 5342 registered pharmacist assistants in Nepal [24], A registered pharmacist is the one registered in Nepal Pharmacy Council after successfully acquiring Bachelors in Pharmacy degree whereas a registered assistant pharmacist is the one registered in Nepal Pharmacy Council after successfully acquiring Diploma in Pharmacy degree. In regard to ART centres, there are 80 centers in 77 districts of Nepal. Among the total participants, 80% (n = 12) the respondents in this study were involved in HIV-related services at some point, and only four of them reported their current involvement in HIV/AIDS-related services in Nepal, implying a figure of only 0.04% involvement.

Importantly, the pharmacists providing HIV-related services are not government employees but are recruited by an international non-governmental organization, Global Fund. This reality was recognized by the participants and they emphasized the importance of expanding their roles and numbers. The primary reason for such a low number of pharmacists in HIV could be due to oversight of government to the ART centers. In addition, our findings showed that the pharmacists in Nepal are currently engaged in the logistics management of the ARVs at the central level. Respondents had a favorable view of pharmacists’ contribution to logistics management. Similarly, the International Pharmaceutical Federation (FIP) has emphasized that pharmacists play a significant role in supply chain management and that their participation can strengthen the medications supply chain [25]. Moreover, the participants agreed on the need to expanding this role to the district, regional and provincial levels (ART centers). Our findings indicated that pharmacists are confined to a central level; nevertheless, given the scopes specified by the respondents, we can assume that pharmacists can make important contributions if major stakeholders intensify their roles and increase pharmacists’ involvement in HIV care.

We inferred that stakeholders see adherence monitoring and pharmacovigilance as potential roles for pharmacists in patient care. While several studies have linked healthcare professionals, including pharmacist-led adherence monitoring to improved treatment outcomes [17–19, 21] pharmacists do not appear to have played an active role in this sector in Nepal. ART has been shown to have clinical benefits for people living with HIV/AIDS; however, poor adherence leads to treatment failure [26]. For successful treatment outcomes, an adherence rate of 95% is recommended [27]. Studies on ARV adherence in Nepal have revealed an adherence rate to ARTs ranging from 66.2% to 94% [28] a figure less than 95% indicating a salient area where pharmacists can contribute.

The pharmacovigilance of ARVs in developing countries is of paramount importance as the toxicity profiles of ARVs in these countries are obscure [29]. PLHIV are reported to take as many as 16 capsules/tablets per day, making them susceptible to many interactions and adverse effects [30], thus calling for the need of continuous monitoring. The WHO guidelines for pharmacovigilance in developing countries have deemed a full-time pharmacist adequate personnel for the role. The participants of this study agreed with this, emphasizing the critical nature of pharmacovigilance in Nepal and identifying it as a potential role for pharmacists. There are fifteen regional pharmacovigilance centers in Nepal that are overseen by the Department of Drug Administration (DDA), in which pharmacists are also involved [31]. Nevertheless, the pharmacovigilance of ARVs is distinct; the NCASC has a separate monitoring and evaluation team that does not include pharmacists.

Pharmacists and other stakeholders held divergent views on the pharmacist’s role in dispensing and counselling antiretroviral medications. This finding can be discussed in the light of several earlier studies. Some have shown ART-related task-shifting from pharmacists to non-pharmacists personnel [32]. Studies conducted in Uganda, Kenya, and Mozambique found positive outcomes where ART was delivered at the community level by non-healthcare professionals or laypeople. Similarly, shifting ART-related tasks from pharmacists to non-pharmacists was found to reduce the burden on patients and Health Care Providers by decreasing long waiting times and frequent visits to ARTs [33]. However, these studies discuss the task shift as a means of relieving pharmacists of their burden. Our study found that pharmacists are underrepresented in HIV care, with all the pharmacists involved in the logistics management of ARVs. This finding matches the Royal Pharmaceutical Society report that states pharmacists’ as the underutilized resource of patient care delivery [34].

Justifying the contrary, pharmacists’ intervention, such as counselling, is reported to ensure benefit to the patients from their therapy [35]. Pharmacists and pharmacies can play an instrumental role in ending the HIV/AIDS epidemic by dispensing ARVs, providing adherence counselling, medication therapy management, pre-exposure prophylaxis, and non-prescription syringe sales [36]. Pharmacists-led counselling has improved outcomes in many acute and chronic conditions, including reducing mortality and morbidity [37].

The findings from our study suggested that there is a lack of awareness among non-pharmacist participants regarding pharmacists’ roles in dispensing and counselling. Such views may be due to the lack of an appropriate dissemination of the messages to the public. A dearth of pharmacists in HIV workforce was mentioned as a major problem. Failure of the policy to subtly define the role of pharmacists can be a possible reason justifying the problem. Additionally, budget constraints were referred as another reason for not recruiting pharmacists. Lack of government support as reflected by non-existent government allotment of pharmacists in the NCASC and ART centers can be an additional factor. Similarly, pharmacist’s own lack of interest and enthusiasm to contribute in the field can be another possible reason.

Our findings indicated that the stakeholders (pharmacists, government, and society) who constitute the entire system were the impediments to pharmacists’ engagement in HIV care. Numerous studies have categorized the barriers to pharmacists’ involvement as pharmacist, government and society related [38–42] which reiterates our findings. A new highlight reflected by our study was the stereotype of pharmacists as ‘manpower’ of pharmaceutical industries; it was held as a chief ground for pharmacists’ lag in HIV care. This conventional thought has been identified as one of the major factors for scantiness of pharmacists entering the HIV care delivery services. There are no government positions available for pharmacists working in HIV-related services and it may be interpreted as the government’s oversight to the importance of pharmacists or the lack of effective lobbying from the pharmacists themselves.

It is evident from our research that there is an understanding among stakeholders regarding the potential role of pharmacists in HIV/AIDS care; yet, this has not been implemented. To overcome the barriers, at least three measures can be recommended based on this study. To begin, policies that clearly define the functions of various health care personnel, including pharmacists should be formulated. Second, governments ought to implement the policies and expand the roles of pharmacists in various ART centers. Finally, there should be self-motivated engagement of the pharmacists.

Self-motivation and organizational support were identified as facilitators of pharmacists’ involvement in HIV care. Pharmacists cited compensation, compassion, and their growth over time as major facilitators. In addition, organizations such as the Global Fund and Save the Children were viewed as facilitators, as these organizations have recognized pharmacists’ roles and hired them for logistic management. Similar to this study, other studies have demonstrated that pharmacists’ involvement in providing care is facilitated by self-motivation, support from fellow pharmacists and the healthcare team, easy access to patients, and organizational support [38, 39, 43, 44]. Our study indicates that lack of the aforementioned facilitators in Nepali context poses a significant barrier to pharmacists’ involvement in HIV care delivery. This study is first to explore the roles of pharmacist in health care in Nepal, and it provides an overview of pharmacists’ activities in HIV/AIDS care delivery. Consequently, this can establish the groundwork for role investigation in various other domains besides HIV/AIDS. On the other hand, this study misses out the perspective of service-receiving end, meaning people living with HIV (PLHIV). Thus, it is possible to undertake additional research that incorporates the patient’s perspective.

## Conclusion

This study explored the current role of Nepalese pharmacists in HIV/AIDS care, scrutinized the barriers and facilitators to their involvement, and identified potential areas for pharmacists to expand their role in HIV/AIDS care. Current situation reflects a dearth of pharmacists in HIV care delivery in Nepal with sheer confinement to the logistic management of ARVs. The pharmacists are reported to be involved only in central logistic management. Dispensing and counselling of ARVs, pharmacovigilance, adherence monitoring, and involvement at regional, district, and provincial levels are the potential roles spotlighted by the participants. Self-motivation, compassion for work, organizational support, growth over time, and the involvement of the Global Fund and Save the Children were identified as facilitators of pharmacists’ involvement in HIV care. Major barriers include stakeholders such as pharmacists, government, and society who constitute the entire system. The finding that the pharmacist and non-pharmacist stakeholders held contradictory views on the potential roles of pharmacists as dispensers and counsellors of ART is of serious concern.. The pharmacists in Nepal have still a long way to go as more barriers than facilitators to pharmacists’ involvement in HIV care exist. Pharmacists thus bear the responsibility to explore and establish their roles in the identified potential areas and contribute to UNAIDS global initiation to end AIDS epidemic by 2030.

## Supporting information

S1 File Interview guide English and Nepali version. (DOCX)

S2 File Approval. Ethical approval from NHRC.
(PDF)

S3 File Interview excerpts. Codebook.
(DOCX)

S4 File Informed consent form English. Sample of informed consent form.
(DOCX)

S5 File Informed consent form Nepali version. Sample obtained from participants.
(PDF)

## Abbreviations

AIDS: Acquired Immunodeficiency Syndrome
ANM: Auxillary Nurse Midwife
ART: Anti-retroviral Therapy
ARV: Antiretroviral
DDA: Department of Drug Administration
FHI360: Family Health International 360
FIP: International Pharmaceutical Federation
GPAN: Graduate Pharmacist Association of Nepal
HIV: Human Immunodeficiency Virus
INGO: International Government Organization
LMD: Logistic Management Division
MOHP: Ministry of Health and Population
NCASC: National Center for AIDS and STD Control
NGO: National Government Organization
NHRC: Nepal Health Research Council
NPA: Nepal Pharmacist Association
NPC: Nepal Pharmacy Council
PLHIV: People Living With HIV
SDG: Sustainable Development Goal
UNAIDS: The Joint United Nations Programme on HIV/AIDS
WHO: World Health Organization
