# Supplementary material for: Potential roles of pharmacists in HIV/AIDS care delivery in Nepal: A qualitative study
Source: PLoS One. 2023 Jan 6;18(1):e0280160. doi: 10.1371/journal.pone.0280160 (PMC9821491; doi:10.1371/journal.pone.0280160)
Supplement: S1 File — (DOCX) [file pone.0280160.s001.docx]

**APPENDIX 1**

**INTERVIEW GUIDE**

**For Pharmacist**

1. Are you involved in HIV/AIDS care? If yes, how long have been involved in HIV care?

तपाईं HIV care सम्बन्धि सेवामा सङ्लग्न हुनुहुन्छ कि हुनुहुदैन? HIV Care प्रदायकको रूपमा काम गर्नु भएको कति भयो?

1. What services are provided to the PLHIV?
   - Who provides these services?
   - What service do you provide?

PLHIV लाई प्रदान गर्ने सेवाहरु के-के हुन्?

- - यी सेवाहरु कसले प्रदान गर्छन् ?
  - तपाइँले के-कस्ता सेवाहरु प्रदान गर्नु हुन्छ?

1. Have you faced any difficulties while providing services as a care provider?
   - Can you tell about such difficulties?
   - Have you felt any factors that have helped facilitate providing service?
   - What changes can be made to facilitate services?
   - Can you tell about your experience as care provider?

एउटा सेवा प्रदायकको रूपमा काम गर्दा, सेवा प्रदान गर्ने क्रममा के-कस्ता अफ्ट्यारो महसुस गर्नु भएको छ?

- - त्यसता कुनै अफ्ट्यारोको बारेमा भन्न सक्नुहुन्छ ?
  - सेवा प्रदान गर्ने क्रमलाई सहज बनाउने के-कस्ता वातावरण यहाँले अनुभव गर्नुभएको छ?
  - यसलाई अझै सहज बनाउन के-कस्ता परिवर्तन ल्याउनुपर्ला अथवा कस्तो कार्यबल थप्नुपर्ला?
  - तपाईंले सेवा प्रदान गरेको कुनै कुराको बारेमा अलिकति आफ्नो अनुभव भन्न सक्नुहुन्छ?

1. According to you, what could be the potential roles of pharmacists in HIV care?

तपाईंको विचारमा Pharmacist को HIV care मा के-कस्तो भुमिका हुन सक्ला ?

1. What is your opinion on pharmacist involvement in HIV/AIDS care?

HIV care मा Pharmacist को सङ्लग्नताको बारेमा तपाईंको के धारणा छ?

1. In your opinion, what impact can pharmacist's involvement in PLHIV care make?
   - What is the impact till now and what can be the impact in the future?

तपाईंलाई Pharmacist को PLHIV care मा सङ्लग्नताले कस्तो प्रभाव पार्न सक्ला जस्तो लाग्दछ?

- - अहिलेसम्म कस्तो प्रभाव पारेको छ र भविष्यमा कस्तो प्रभाव पार्नसक्छ?

1. How the current pharmacist's involvement does compares to the past?
   - In what aspects has it been easy?

पहिल्यैको तुलनामा हाल Pharmacist हरुको सङ्लग्नता कस्तो देखिन्छ ?

- - कुन कुन कुरामा अहिले सजिलो भएको छ?

1. Who handles ART related dispensing, counselling and adherence monitoring in the context of Nepal?

नेपालको सन्दर्भमा ART संबंधित dispensing, counseling, adherence monitoring जस्ता गतिविधिहरु कस्ले गरिरहनु भएको छ?

1. In your opinion, are pharmacists necessarily required for ART related activities like dispensing, counselling and adherence monitoring, or, it makes no difference if other healthcare workers handle these activities?

तपाईंको विचारमा ART संबंधित dispensing, counseling, adherence monitoring जस्ता गतिविधिहरुमा Pharmacist कै सङ्लग्नता जरुरी छ कि अन्य स्वास्थकर्मीहरुले गर्दा पनि फरक पर्दैन?किन?

1. Should pharmacists explore their role as a HIV care provider?

Pharmacist हरु आफैंले HIV Care प्रदायकको रूपमा आफ्नो भुमिकाको खोजी गर्नु पर्छ कि पर्दैन?

1. What should be done differently or what steps should Pharmacists take to make their place in HIV care delivery in future? What do you want to suggest to pharmacists?

भविष्यमा HIV care delivery मा आफ्नो ठाँउ बनाउन अहिले भन्दा के फरक गर्नुपर्छ वा कस्तो कदम उठाउनु पर्छ? Pharmacist हरुलाई के सुझाव दिन चाहानुहुन्छ?

**APPENDIX 2**

**For Policy makers**

1. What kind of services does your organization provide relating to HIV care delivery?

- How many staff are involved?
- What number of pharmacists are involved?
- Are they enough?

HIV care delivery सम्बन्धि तपाईंको संस्थाले के-कस्ता कामहरु गरिरहेको छ?

- - कति कार्यबल छन्?
  - यस् संस्थामा कतिजना Pharmacist कार्यरत हुनुहुन्छ ?
  - के यति पर्याप्त छन् ?

1. In your opinion, is the involvement of Pharmacists necessary in PLHIV care?
   - Why?

तपाईको विचारमा PLHIV care मा Pharmacist को सङ्लग्नता आवश्यक छ कि छैन ?

- - किन?

1. What does the involvement of pharmacists in HIV care delivery look like in other countries? And what does the involvement of pharmacists look like in Nepal?
   - What could be the reasons for this?

HIV care delivery मा अन्य देशमा Pharmacist हरुको सङ्लग्नता कस्तो देखिन्छ? र नेपालको सन्दर्भमा Pharmacist हरुको सङ्लग्नता कस्तो देखिन्छ?

- - यसका कारणहरु के-के हुन सक्छन् ?

1. Do you have any policy regarding HIV care delivery for Pharmacists in Nepal?
   - If yes, what are the provisions? What are the policy rules?
   - Have those policy rules helped or not?

नेपालको सन्दर्भमा Pharmacist हरुको HIV care delivery सङ संबंधित कुनै Policy छन् कि छैनन्?

- - छन् भने के-कस्ता प्राबधान हरु छन्? कस्ता नीति नियमहरु छन् ?
  - ती नीति नियमहरुले सहयोग गरेको छ कि छैनन्?

1. Who handles ART related dispensing, counselling and adherence monitoring in the context of Nepal?

नेपालको सन्दर्भमा ART संबंधित dispensing, counseling, adherence monitoring जस्ता गतिविधिहरु कस्ले गरिरहनु भएको छ?

1. In your opinion, are pharmacists necessarily required for ART related activities like dispensing, counselling and adherence monitoring, or, it makes no difference if other healthcare workers handle these activities? Why?

तपाईको विचारमा ART संबंधित dispensing, counseling, adherence monitoring जस्ता गतिविधिहरुमा Pharmacist कै सङ्लग्नता जरुरी छ कि अन्य स्वास्थकर्मीहरुले गर्दा पनि फरक पर्दैन? किन?

1. Should pharmacists explore their role as a HIV care provider?

Pharmacist हरु आफैंले HIV Care प्रदायकको रूपमा आफ्नो भुमिकाको खोजी गर्नु पर्छ कि पर्दैन?

1. What should be done differently or what steps should Pharmacists take to make their place in HIV care delivery in future? What do you want to suggest to pharmacists?

भविष्यमा HIV care delivery मा आफ्नो ठाँउ बनाउन अहिले भन्दा के फरक गर्नुपर्छ वा कस्तो कदम उठाउनु पर्छ? Pharmacist हरुलाई के सुझाव दिन चाहानुहुन्छ?

**APPENDIX 3**

**For policy executors**

1. Are you involved in HIV/AIDS care? If yes, how long have been involved in HIV care?

तपाईं HIV care सम्बन्धि सेवामा सङ्लग्न हुनुहुन्छ कि हुनुहुदैन? HIV Care प्रदायकको रूपमा काम गर्नु भएको कति भयो?

1. What services are provided to the PLHIV?
   - Who provides these services?
   - What service do you provide?

PLHIV लाई प्रदान गर्ने सेवाहरु के-के हुन्?

- - यी सेवाहरु कसले प्रदान गर्छन् ?
  - तपाइँले के-कस्ता सेवाहरु प्रदान गर्नु हुन्छ?
  - Pharmacist ले के-कस्ता सेवाहरु प्रदान गर्नु हुन्छ?

1. In your opinion, is the involvement of Pharmacists necessary in PLHIV care?
   - Why?
   - What impact can the involvement make?

तपाईको विचारमा PLHIV care मा Pharmacist को सङ्लग्नता आवश्यक छ कि छैन ?

- - किन?
  - सङ्लग्नताले कस्तो प्रभाव पार्न सक्ला जस्तो लाग्दछ?

1. Who handles ART related dispensing, counselling and adherence monitoring in the context of Nepal?

नेपालको सन्दर्भमा ART संबंधित dispensing, counseling, adherence monitoring जस्ता गतिविधिहरु कस्ले गरिरहनु भएको छ?

1. In your opinion, are pharmacists necessarily required for ART related activities like dispensing, counselling and adherence monitoring, or, it makes no difference if other healthcare workers handle these activities? Why?

तपाईको विचारमा ART संबंधित dispensing, counseling, adherence monitoring जस्ता गतिविधिहरुमा Pharmacist कै सङ्लग्नता जरुरी छ कि अन्य स्वास्थकर्मीहरुले गर्दा पनि फरक पर्दैन?

- - किन?

1. According to you, what could be the potential roles of pharmacists in HIV care?

तपाईंको विचारमा Pharmacist को HIV care मा के-कस्तो भुमिका हुन सक्ला ?

1. Should pharmacists explore their role as a HIV care provider?

Pharmacist हरु आफैंले HIV Care प्रदायकको रूपमा आफ्नो भुमिकाको खोजी गर्नु पर्छ कि पर्दैन?

1. What should be done differently or what steps should Pharmacists take to make their place in HIV care delivery in future? What do you want to suggest to pharmacists?

भविष्यमा HIV care delivery मा आफ्नो ठाँउ बनाउन अहिले भन्दा के फरक गर्नुपर्छ वा कस्तो कदम उठाउनु पर्छ? Pharmacist हरुलाई के सुझाव दिन चाहानुहुन्छ?
