# Supplementary material for: Potential roles of pharmacists in HIV/AIDS care delivery in Nepal: A qualitative study
Source: PLoS One. 2023 Jan 6;18(1):e0280160. doi: 10.1371/journal.pone.0280160 (PMC9821491; doi:10.1371/journal.pone.0280160)
Supplement: S3 File — Codebook. (DOCX) [file pone.0280160.s003.docx]

|  |  |  |
| --- | --- | --- |
|  | Organizations role   - Funding - Global fund - Procurement - Coordination with government - Grant monitoring - Supply chain - Strengthening government - Commodity procurement - One door policy | - *KI04: Save the children comes under the global fund. The global fund is a donor organization that supports HIV, TB, and malaria. Its principal recipient is Save the children that means the fund initially comes to Save the children.* - *KI05: "I started working from UNDP HIV Nepal, a Global fund supported program, supporting NCASC. The main role of UNDP HIV is procurement and supply of ARVs, commodities, grant monitoring, and supporting NCASC. Currently, Save the children is the execution organization of Global fund in Nepal. So save the children is helping MOHP."* - *KI04: The roles of Save the children are: Implementing HIV program in cooperation with the government, Making strategy and coordinating it with the government, Grant monitoring, Strengthening government, HIV treatment care, and procurement.* - *KI03: "HIV/AIDS is funded by Global fund. Save the child is the principal recipient (PR). The roles of STC are: Procurement of medicines, test kit, lab reagents, equipment, and workforce handling* - *KI07: “Later around 2010-2011 Global fund came intending to make the government capable, so they handed it over to the government. The distribution system was supposed to be looked over by LMD through one door system. Later, when it was handed over to the government, they did not create a post for pharmacists but the support of Save, the children, recruited pharmacists and then they started procurement process.“* - *KI01: “. Till now, there is one door policy meaning NCASC supplies directly. If you see, in other diseases such as TB, malaria the medicines are sent from the centre to the region and then to the district, but, in the case of HIV medicines, they are distributed directly from the centre, i.e., NCASC to the ART centres. “* |
|  | - Number of pharmacist - No pharmacist through government quota | - *KI03: "There are two sides in which HIV is looked after. First through the Save the Children country office and second through the pharmacist stationed at the government by Save the children. There are… wait.. 1… 2 pharmacist in government, i.e., NCASC and 2 in-country office."* - *KI12: "Unfortunately, there is no pharmacist in NCASC. There are 2 pharmacists, and they are from Save the children; other than that, we do not have our pharmacists."* - *KI04: Talking about the Global fund team, there are 1, 2, 3…..5 pharmacists now. We do not only look after HIV. Under the global fund country office, 2 work in HIV, 1 in TB, 1 in malaria, and 2in NCASC. So all together, 6 pharmacists in Global fund of which 4 in HIV.* |
|  | Existing status   - No pharmacist in ART centers - Current number of pharmacist - No involvement - Number of ART centers | - *KI12: "Unfortunately, there are no pharmacists in the ART centers* - *KI06: There are pharmacists' in NSASC and a supporting agency to save the children where I worked. However, there are no pharmacists in general logistics in regional medical store and provincial medical store or below that level."* - *KI04: "There are no pharmacists in ART centers till now. For instance, in Palpa Mission hospital, an ART clinic but the pharmacist is working there do not* *directly support the ART centre..so..um.. there is no direct role, but they support.“* - *KI01: “From the beginning, there has been no involvement at the site level, but only at the central level, so now they are trying to create involvement at the province level."* - *KI01: "There are 79 ART centres. Talking about the number of pharmacists, there are 2 of us in the main level at NCASC.* - *KI12: There are 80 ART centres.* |
|  | - Dispensing and counselling by others | - *KI12: "For dispensing and counselling, we have our ART counsellors in every centre. Pharmacists are not directly involved there."* - *KI06: Dispensing and counselling are done by HA, the staff nurse. They are trained.* - *KI01: "PCL nursing dispenses. At some centres, there is Bsc. Nursing, BN, or those with public health degree…and in some places, there is HA. However, maximum there are either HA or staff nurse."* - *KI11: "Actually… all the service centres… where there are HIV ART centres or service units..they are also called SDU. So the personnel working there are trained.. they are given awareness training, and they are either from nursing background or public health background, but there are rarely people with a pharmacy background. To my knowledge, there are very few."* |
|  | - Expert in medicine - Importance of pharmcists - Supervision - Pharmacists knowledge | - *KI14: "Others are not allowed to dispense or counsel. It is illogical if staff nurse counsel, there should be a pharmacist because..um.. say I am an expert in a certain area and when I give my opinion on that matter I give it through the in-depth knowledge that I have. If you are not an expert, you might know what to tell the patient, but you will not know why to tell or the reason behind it, and if there are any consequences, you might not be able to handle it. So, pharmacists have in-depth knowledge, and they can counsel patients accordingly."* - *KI13: "We study about the medicines, so what is the role of the pharmacist? When we say pharmacist, we mean medicine expert. So, dispensing and counseling is the role of the pharmacist. If it is done by HA or staff nurse, that is okay, but it should be done under the supervision of the pharmacist."* |
|  | - Positive impact - Proper counselling by pharmacist | - *KI01: "Pharmacist can look after dispensing in the ART centres. The pharmacist can deliver far more and better facilities to the patients than they get through HA and staff nurse dispensing. We tell this at sites as well. The impact pharmacists can make firstly would be convincing the patients to adhere to the medications via counselling like medicines should be taken regularly, not skip the medications, and the side effects and adverse effects. If a staff nurse does not know that medicine might have a side effect, she cannot tell the patient, but as pharmacists, we know about it, and we can make a difference. For instance, Nevirapine syrup induces nightmares for the first 14 days of administration. So, if patients are not counseled on this, they might stop the medicine, but if they are counseled, then they will be prepared for it."* - *KI07: "It would be better if dispensing, and the pharmacists do counseling. Extensive counseling is needed for the patients regarding when and what amount the medicine should be taken. For example, there was a case where a patient used to administer the tablet by breaking it, so when I found out that, I immediately told that it should be taken whole; otherwise, the efficacy might be lost. The patient should be properly counseled, so if pharmacists are involved, it would be better."* - *KI05: “Pharmacist can properly counsel regarding the time and manner of administration. Along with that, they can dispense. If patients are properly counseled, they follow the instruction. So, that particular manner of counseling and timing of counseling is critical which a pharmacist can deliver."* |
|  | - Well-placed - Pharmacists knowledge - Impact of pharmacist | - *KI11: "Those who have knowledge and skills in dispensing and are well-known about the medicines, they should do it for the better treatment outcome. Pharmacists are well placed because they are given training since bachelor's degree, and they know medicines. They know about the effect of medicines, and they can give information required by the patient. Moreover, because of this, the treatment outcome will be positive. However, in this very matter or subject, if a specified person is well-trained, then we cannot argue that the person cannot perform well, but certainly, pharmacists are well-placed.“* |
|  | - Basic requirement | - *KI06: "In GDPs, for now, if not pharmacist, at least diploma pharmacist or those who are trained on GDPs should be involved. However, as far as the program is concerned, pharmacists are needed."* - *KI07: "At least assistant pharmacist should carry out dispensing, storing, and patient counselling."* |
|  | - No need of pharmacist - Not pharmacist roles | - *KI09: "This role is different. This role belongs to monitoring, evaluation, treatment, and care, and I do not think this is pharmacists' role."* - *KI09: "Pharmacists…. in one way, how they assure whether the medicine has been administered or not.. that is different, and I do not think the government has thought in that way. So maybe there is lacking, or it is because there is no importance of pharmacists there, but in my understanding, that is not the role of the pharmacist."* |
|  | - No visible roles | - *KI08: "Till now, there are counselors plus staff nurses or those with a medical background in the programmatic approach. So dispensing. um.. because when the patient comes, they do the counseling, so they also dispense. In ART centers, the patient volume is not that significant, so there is no need for the pharmacist. Furthermore, I do not think pharmacists counsel the patient, and when the patient comes to the ART center, recording, reporting of their status should be done so, nurses do that. About counseling… I think, most probably, counseling can be done only by the trained counselors. Pharmacists…if you see in japan doctors dispenses the medicines so, they do not even dispense in japan. So, different countries have different contexts, and in our country, staff nurses and HA do the counseling because they have the specific background and are the ones to look at these matters after doctors.“* |
|  | - Budget/ no visible roles - Importance of doctors | - *KI12: There are 80 ART centres, and we have not been able to retain even doctors in all of them because of budgetting issue, so, lets not speak about others. So, one there is budgeting, and another there is no role visible role of the pharmacists.* |
|  | - Procurement and supply chain - Quantification - Forecating - Quality Aussuarance - Training - Pediatric dose calculation - Technical specification - Procurement/commodities - Modality of procurement and supply - Warehousing - pharmacist knowledge on proper storage conditions - Supportive role | - *KI05: " There are about 3-4 pharmacists in Nepal, and their main role is procurement and supply management. Now, they are slowly supporting in strengthening the supply chain. There they are properly ensuring from procuring commodities to storage, warehousing, distribution, and delivery up to the last mile."* - *KI04: "My main role is procurement. We look at the whole part of procurement, from distribution to all."* - *KI12: "Currently under medicine plan pharmacists look after procurement planning, supply chain, pipeline monitoring, quantification, and forecasting. Pipeline monitoring or checking means to ensure the smooth run..without any problem from procurement to the supply plus the recording and reporting."* - *KI08: "I have a public health background. I am not a pharmacist, but my colleagues who were pharmacist their focus was basically on forecasting and quantification."* - *KI07: "I used to look after forecasting. So, as per the existing regimen, how much, what quantity or what item of medicines are required, and then, according to program the forecasting as per tentative cases is done, for instance, there are records of cases like how many children and adults are there this year and, as per those data firstly required medicines are calculated and then, as per calculations the procurement process is arranged."* - *KI09: "Our pharmacist friends they request for procurement. In order to procure, we ask for technical specifications for medicines, reagents. So, they prepare technical specifications..um..like how is the market, how is the availability like… so they study the market and give us a report and then based on that report we move forward.* - *KI02: "We have a general evaluation committee and a technical committee. Currently, there are a total of 4 pharmacists in a technical committee where we prepare technical specifications as per the need, and we monitor the adherence to the specification."* - *KI01: “one, we support the procurement process. Another, the country-wide supply chain, supply of the commodity, there is ARV medicine, then, test kits, RDT, and reagents for viral load and CD4. Similarly, the supply of harm reduction tools like condoms, lubricants are also supplied from here, and we look over the country-wide supply chain of the syringe.“* - *KI02: “There are more than 200 commodities. We supply ARV drugs, RDT, test kits, STI drugs and, we keep records."* - *KI02: "We work on the part of the supply. We have different sites throughout Nepal. We have this system; it is called bimonthly supply, where we supply commodities every 2 months.* - *KI01: "We look over procurement. There is tender bidding like we have to procure annually. We procure medicines required for 23-24 months.* - *KI02: "We manage the warehouse. In warehouse. As a pharmacist, we know how to know right, like we have to store commodities at different temperatures."* - *KI03: "There are different divisions, for instance, those in entities, they ensure supply chain of pharmaceutical and health product, avoid stock-out, then monitor adherence to the treatment guidelines, whereas, as a part of the country office, what I do is monitor them plus look after the need of the government and the availability of the commodities required by the government, then, ensure whether the government will be able to support what it procures. So, the roles are different. Roles differ with the designation."* - *KI05: "When we look at the HIV program in Nepal, since the beginning, i.e., at least 12-14 years pharmacists have been involved. Our role or our job is not directly clinical, but is the procurement of HIV-related drugs, their successful storage and distribution as per schedule to the ART centers in order to avoid stock-out and under-stock."* - *KI04: "Another role of the pharmacist is Quality Assurance and Quality control. We mainly look for certain standardized medicines, which are our wish and the donor's wish. The donor says that we cannot implement the program without a certain level of standardized products, so we ensure procurement from at least the WHO-GMP-certified manufacturer site. It means that, at least when the medicine reaches the client, it is of a certain level."* - *KI05: "How to buy quality products? There are certain guidelines, and we have to meet all the quality parameters to secure the quality product. If you follow the guidelines, then you will be sure about the quality of the products."* - *KI02: "During procurement, we look over quality assurance and technical evaluation. As a technical pharmacist, we ensure WHO prequalification of medicines. After receiving medicine, we put them in quarantine, and then we carry out the technical evaluation. In technical evaluation, what we do is, we physically examine the medicine and we also look after the provided certificate of analysis."* - *KI09: "After procurement, we ask the pharmacists to do the technical evaluation like post-inspection of the goods. Like when the goods arrive.. we buy medicines, reagents, test kits so in order to inspect them we have designed a format like WHO-PQ, so they check whether the goods are up to the standards."* - *KI03: Talking about my role, one of my roles is procurement and supply chain management, and the other is to strengthen the government in terms of procurement and supply of medicines and health products. In this, we equip the government, give training regarding specifications, quality assurance. So these are the parts I am overseeing. Sometimes, we calculate pediatric dose.* - *KI04: We also give logistic training …. We provide support to ART centres. For instance, sometimes they reach out to us regarding the pediatric dose, so we calculate the dose based upon the age and body surface area and get back to them.* - *KI05: " There are about 3-4 pharmacists in Nepal, and their main role is procurement and supply management. Now, they are slowly supporting in strengthening the supply chain."* - *KI12: "Problems keep coming from the district. Even though we all tackle the problem together, they are the main ones. They are the first contact."* |
|  | - No advocacy - GPAN - NPA - NPC - DDA - Lack of pharmacist - Need of pharmacist - Professional weakness - No effort | - *KI04: "I do not think there are pharmacists involved everywhere in Nepal. We see the need for pharmacists in many areas, especially in district-level pharmaceutical storage. There is no pharmacist there till now. Pharmacists' post has been fixed in regional warehouses, but it would be better to allocate pharmacists in the lower-level warehouses where you are supposed to handle the medicines. Nevertheless, in the context of Nepal, that system has not been followed yet. We need to advocate for it. Mainly, it is due to the professional weaknesses that we have not been able to change the system. We have not been able to put forward our voices to the authority. I do not think our professional organizations like GPAN, NPA have been working on that level."* - *KI05: "Currently, nurses are challenging doctors. They are fighting for their roles, but pharmacist associations have not been able to impact them. There is a lack of connective roles. For instance, if a pharmacist has an idea that he cannot do anything independently, he needs a platform. Platforms are our associations like GPAN, NPA but, I hardly find them helpful. My seniors established GPAN, I think around 1994 or 95, but I do not see their role. Similarly, we have NPC, NPA. You can approach individually, but there is no integrated effort."* |
|  | - No lobbying/ aaadvocacy - Policy level weakness - No pharmacist | - *KI03: "The reason why pharmacists are not in ART centers is because of weaknesses in the policy level, meaning those among our professionals who are involved in policy level, it is because of their weaknesses. It is because of the lack of lobbying. Advocacy should be done from a high level. One such hub is DDA, and another is NPC. I do not see NPC advocating in the current scenario. I have told them as well because it is something they have to do. NMC has been strongly advocating for doctors.* - *Similarly, public health associations have also reached the policy level, but why can't the pharmacy council do so. They are the ones responsible for our professional advocacy. I am not sure whether they are unable or unwilling to do so."* - *KI12: "There was no pharmacist topic previously, so this is new. Maybe it is because there are no pharmacists. You should not only be limited to organograms. Furthermore, secondly, it is because of insufficient advocacy. Maybe you have your professional association or organizations, so you have to increase your self-value through them. I do not know if they are active, but the association must advocate for their roles. We feel the need of the pharmacist, so we often advocate, but there is no such advocacy from that side."* - *KI11: "Our profession is a developmental phase. We have pharmacist associations and different kinds of organizations, and these organizations have failed to advocate for upbringing our profession, to develop and increase our professional capability. For example, an organization that advises in policy-making like there is an organization called a council. Moreover, these organizations do not function in this, but rather they play professional politics, self-politics. So, they have been fulfilling their professional and communal politics, but professional advocacy is lacking."* |
|  | - Self-limited scope - Industrial focused - Unwillingness to explore roles - Unawareness - No focus | - *KI14: "In the context of Nepal, pharmacists' are more vital in the industrial side, and few of them are going to the hospital, but the clinical part is untouched."* - *KI05: "Instead of exploring their roles, pharmacist they follow the traditional pathway in Nepal. As soon as someone finishes their degree, they go straight to the manufacturing company. This was the scenario when I was in Nepal. Even my friends went to manufacturing, and it gradually saturated, and then, they started shifting towards the hospital and then marketing. So, there are lots of new fields which we can be uncovered. We have medical research. So, we have to advocate."* - *KI01: "In Nepal, almost all pharmacist are pursuing their career in manufacturing company, hospital pharmacy or marketing. They work in multinational or Nepali companies, but I think no one is interested in the supply chain. Pharmacists have not yet understood the supply chain. They are unaware of its scope. So, one, we are unaware, and another, we have not yet focused there."* |
|  | - Unwillimgness to explore roles - No research base - Busy in day to day work - No self initiation | - *KI12: "Pharmacists should look over medicine storage, distribution, and research regarding the effectiveness of new medicines, but in the case of Nepal, there is no research base. Here, they are just procuring and distributing, only doing what they are asked to do, so there is no self-initiation. So I would say there is no research base at all. So they should explore their roles rather than only doing what they are asked."* - *KI03: "There is no such demotivation factor but only unwillingness among pharmacists that is holding them back. This is what I see in the current scenario. One, pharmacists themselves being unable to make others understand their part, and second, they are not moving forward. Pharmacists are busy with day-to-day routine work. They are not exploring their role, which is why their role is not seen. Earlier, there was one Pharm D. in NCASC. I will not take his name, but he could not establish himself in the clinical part. So, I think that you should be able to show your role. Moreover, we do not have a solid research base. Those who have gone to foreign countries only have built a solid research base. "* |
|  | - Lack of pharmacists - Not enough pharmacists | - *KI06: "The reason of pharmacist not flourishing in HIV is firstly the number, and another is the ongoing practice. If it were in practice from the beginning, then the scenario would have been different. This is because of lack of pharmacist firstly, and secondly, pharmacists are more involved in manufacturing companies."* - *KI12: "There was no pharmacist topic previously, so this is new. Maybe it is because there are no pharmacists. There are no pharmacists in our sector, but we need them. So, I have sent a letter to the government mentioning that if the government cannot reform, then at least provide us with pharmacists on a two-year contract basis. Let us see what happens."* - *KI01: "Have you heard of a pharmacist working in WHO? There were none then. I worked there in the supply chain for about three years and, now again, there are none. There are no pharmacists in UNICEF as well, but save the children has hired pharmacists* |
|  | - No government allotment - No posting | - *KI02: "Talking about policy level, even in NCASC there is no allotment for pharmacists' from the government. So, most of the time, especially during supply, we have to communicate with people of different backgrounds, and it is challenging to make them understand. So, it would be easy, especially in the supply chain, if more pharmacists were involved.* |
|  | - Lack of understanding - Unaware government - Policy level weakness | - *KI01: "In other countries, pharmacists are involved in HIV on a micro level, but unfortunately, that is not the case in Nepal. I think this is because of lack of understanding from a policy level."* - *KI05: "Pharmacists are not engaged in dispensing practices, and there is no government posting of pharmacists. There are 2-3 factors for this. One, lack of awareness. Second, there is no such vision or focus from government or say policy level and, third, they are struggling with managing the existing sources in the health system.“* |
|  | - Biasedness - Domination - Doctor dominative | - *K103: "There is no government posting meaning there should have been a permanent post for pharmacists, but that is not the case. Firstly, I think the Ministry of health, or the whole government, has not understood that well, and secondly, the policymakers are a bit biased. Let us say if I were in that authoritative position then, I would prioritize my profession only. So, that has been the trend. What I mean is, earlier, doctors were abundant at the policy level, and during that time, they only focused on their development and did not care about other faculties. Similarly, these days' public health administrators seem to have their foot in the policy level, doing the same. I think public health is an umbrella that embodies pharmacy as well. We are a part of public health. So, they should have taken an integrative approach and flourished every aspect of the public healthcare team equally, like creating a post for every health profession. However, I do not see that happening."* - *KI05: "Doctors have been dominating since the beginning. So, what happens when doctors dominate at the policy level? So, firstly there were few pharmacists and more doctors, and it is a basic human nature that when you have been dominating someone then, you always want to keep up."* - *KI06: "If you compare to the past, there is no drastic change. The number of pharmacists has increased with the number of HIV programs, but it was a doctor's dominative area and is still the same."* |
|  | - Biased system - Equal roles | - *KI15: "There seems dominancy in the health care system. Public health personnel, nursing staff, and physicians are more involved than the pharmacist. There should have been pharmacists' role along with their roles."* - *KI14: "The healthcare system in Nepal seems to be somewhat biased like they consider doctors to be an important person and have been unable to integrate other sectors. This continuation has not been broken yet. Maybe it will break slowly in future and go in a teamwork approach."* |
|  | - No social regonition - Societal unawareness - Doctor dominative - Need to prove | - *KI15: "In the context of Nepal, we have not gotten social recognition yet."* - *KI06: "Generally, our society or community are still unaware of the arrays of services we are capable of providing. They follow doctors and what you see now is the reflection of the whole society."* - *KI05: "The reason why pharmacists are lagging is, one, initially there were not many pharmacists, and second, doctors interact with the patients directly; I mean face-to-face. So, once the patient has a direct interface, they feel the importance of doctors, but they fail to see those who support the doctors and do not even care about it. Since doctors become the face, they diagnose and prescribe, but pharmacists manufacture that medicine, nurses take care of them, and other supporting staff. The patient only sees the doctor. uiThis has been in practice since the very beginning. Doctors have been dominating.“* - *KI13: "The challenge is that we have to prove ourselves in the country and in the society time and again. As per the drug act, a 48-hour trainee can also distribute the medicine, so we have to prove that we are better than them."* |
|  | - Outdated policy - Not defined roles - No specific policies | - *KI02: "Till now, there is the policy where ARV dispensing and counselling are said to be done by 5th level officers like PCL nursing and HA. It has not been updated yet."* - *KI01: "Dispensing, counselling is done by staff nurses and HA. They made this policy, I guessed around ten years ago, and, at that time, they had no idea about the need for pharmacists. However, now we are raising issues regarding this. We have been telling where there is medicine. There should be a pharmacist."* - *KI04: "There are no specific policies."* - *KI02: "We have not been able to make an impact on the clinical side because of the policy constraints. It is all up to the policy and policymakers to decide, and if they think pharmacists are needed in the clinical part, it is possible. However, that has not happened till now."* - *KI05: "If you go to the website of DDA or certain MOHP pages, then you will see certain guidelines regarding the need of pharmacist in hospital pharmacy, storage, dispensing practices but, there are no HIV specific guidelines. At least there were no such specific guidelines when I was in Nepal, and I do not think there is one now."* |
|  | - Importance of new policies - Clear policies - Defined roles - Proper roles assignment - Integrated policy | - *KI07: "There are activities related to procurement and then specifications of medicine and other commodities and …then different parts quality control. Similarly, there are many parts, and these parts need to be specified as the role of pharmacists, but that concept is not developed."* - *KI15: "I believe that instead of a new policy, an integrated policy should be made, and our roles should be specified."* - *KI14: "If roles are clearly defined, everyone's space is made clear, and roles are assigned per expertise in the existing health structure of the nation, then the biasedness will gradually minimize.“* |
|  | - Biased policy - Doctor-oriented policy - Public health oriented policy - Need to explore roles | - *KI05: "In the context of Nepal, health means doctor. This is the conception till now but, health does not mean doctor. Doctors are one of the components of health. There is this big chain of professionals that holds the chain. There are nurses, pharmacists, paramedics, and other staff as well. However, since the beginning, everything is doctor-focused in the health sector, and pharmacists are lagging. Still, health means doctors and policies are also made accordingly."* - *KI02: "If you see the Ministry of Health policy, it is more Public health-oriented. So, we have to fight at policy level for our roles but, first, we have to explore our roles and be capable to assume those roles."* |
|  | - Organogram - No implementation - lack of structural requirements - Structural problems | - *KI12: "I have heard that there is the provision for pharmacists' involvement in the policy, but that has not been reflected in the organogram."* - *KI11: "There is the policy in the drug act relating to the encouragement of medicine-related usage, but if we talk about policy guidelines, in the health policy, there is the provision to ensure that adequate and qualitative health services are provided. So, both of these policy guidelines state that you should be capable of providing quality health services. So, any professional services are required to ensure that the services are effective, qualitative, and suitable to the patients. Because of this, there is no problem with the policy. Now, talking about the position and structure at our operational level, it is not in structure. Do you understand what I am trying to say? Structure.. There is a lack of structural requirements. For example, there should be a position for pharmacists where patients are treated, but the allotment arrangement could not be made, which is why there is an insufficient workforce leading to pharmacists being unable to provide services. So, a policy is not restricting, but the structure is creating problems.* - *KI11: Structure is made by the health ministry and Nepal government altogether. Health ministry has to propose. The related body determines the structure from a general health post to the tertiary care hospital and different departments. In this case, health ministry should propose, and that is lacking."* |
|  | - NGO - INGO - Global fund - Personal satisfaction - Realization of importance - Self improvement - Salary | - *KI04: "I believe it is satisfying to be involved as a pharmacist. Other than that in this NGO, INGO level the salary is also a motivational factor, and lastly exposure."* - *KI01: "I feel happy to see the patients get the medicine on time like yesterday, medicines arrived, and we supplied. There is no such thing as mine or yours here, and our main focus is that patients should get medicines on time. If we do not supply, there might be stock out in the district, and patients will not get the medicines. So, I feel happy in that aspect.“* - *KI05: "As I started working, people began to realize my roles and importance. Before that, no one knew my capabilities, but as I kept working, I gave results that were appreciated by my supervisors, seniors….and the whole organization. Only then, they realized he could do this, and he is a very efficient employee. So we have given results which is why nowadays they have started hiring pharmacists."* |
|  | - Global fund - Save the children - Pharmacist recruit,ent - Pharmacists involvement | - *KI05: "Global fund operates throughout the world, and since its operation in Nepal, people have come to know about the pharmacist especially their need in drug sector and distribution sector. This has led to many organizations hiring pharmacists' especially in drug distribution and procurement."* - *KI02: "In other countries, pharmacists are widely involved in HIV, which is why Global fund hired pharmacists.. they brought that concept in Nepal. Earlier, there was no such practice in Nepal. Since, Global fund started recruiting pharmacist..um..similarly, USAID and then WHO..when these organizations started to recruit pharmacist, then everyone realized the need of pharmacist."* - *KI07: "The distribution system was supposed to be looked over by LMD through one door system. Later, when it was handed over to the government, they did not create a post for pharmacists but the support of Save the children recruited pharmacists and then they started procurement process."* |
|  | - GPAN - NPA - NPC positive role - Professional organization as facilitators | - *KI13: "There are different organizations like GPAN, NPA. GPAN has not been able to play that much of the role. I do not see their role. DIPSAN for the diploma is rather coming aggressively. There should be unity amongst the pharmacist. There is Nepal Pharmacy Council… it is playing a positive role, and there are many other INGOs who are hiring pharmacists' to handle medicines."* - *KI15: "There are different INGOs and NGOs. Similarly, there is NPC. If any such proposal comes to the pharmacy council, I am ready to take it to the board, if necessary, to the Ministry of health as it is our line ministry. So, to discuss and communicate or set up a meeting with MOHP, I am the contact person here, and I am ready."* - *KI14: "Our professional organizations are to support the pharmacists. NPA is also a facilitator, and we have been working for the pharmacists."* |
