# Supplementary material for: Potential roles of pharmacists in HIV/AIDS care delivery in Nepal: A qualitative study
Source: PLoS One. 2023 Jan 6;18(1):e0280160. doi: 10.1371/journal.pone.0280160 (PMC9821491; doi:10.1371/journal.pone.0280160)
Supplement: S4 File — Sample of informed consent form. (DOCX) [file pone.0280160.s004.docx]

**INFORMED CONSENT FORM**

Namaste, I am Ayushma Shahi, an M.Pharm student at Kathmandu University. As part of my academic curriculum, I am conducting research titled "**EXPLORING THE ROLES OF PHARMACISTS IN HIV CARE DELIVERY IN NEPAL."** The main aim of my study is to explore the roles of the pharmacist in HIV care delivery in ART centers, determine the existing pharmacy workforce involved in HIV care delivery, and identify the perceived facilitators and barriers to pharmacists' involvement in HIV care. For this purpose, I have to ask a few questions following the interview guide. The information provided by the respondents shall be maintained with the utmost confidentiality, and their identification shall not be disclosed. Furthermore, no financial transaction shall be made, and your participation in this study shall be voluntary. Please read the statement below.

**Time required (approx.):** 30-60 minutes

**Statement:**

1. I understand that my interview may be recorded.
2. I understand that data collected during the study may be looking at by other researchers and regulatory authorities.
3. I understand the reasons for this interview and am willing and happy to participate in it.
4. If I agree to participate in this interview, I understand what I will be required to do.
5. The interview I give and the information it contains will be used solely for the purposes defined by the project.
6. I know that I have the right to leave the interview at any time or to refuse to answer any questions.
7. If I do not agree to participate in this interview, I understand that I will not be penalized by the researchers nor by any medical service personnel in the future.
8. I voluntarily agree to participate in this interview.

…………………………………………………………. ……………………………………………….

Signature Date
