# Supplementary material for: Potential roles of pharmacists in HIV/AIDS care delivery in Nepal: A qualitative study
Source: PLoS One. 2023 Jan 6;18(1):e0280160. doi: 10.1371/journal.pone.0280160 (PMC9821491; doi:10.1371/journal.pone.0280160)
Supplement: S5 File — Sample obtained from participants. (PDF) [file pone.0280160.s005.pdf]

सूचित सहमति फारम

नमस्ते, म आयुष्मा शाही, काठमाडौं विश्वविद्यालयको एम. फार्म विद्यार्थी हुँ। मेरो शैक्षिक पाठ्यक्रमको अंशको रूपमा, " PREDICTORS OF KNOWLEDGE, ATTITUDE, PRACTICE AND ADHERENCE OF ANTIRETROVIRAL THERAPY AND QUALITATIVE INSIGHT OF POTENTIAL ROLES OF PHARMACISTS IN HIV/AIDS CARE DELIVERY" शीर्षकको अनुसन्धान गर्दै छु। मेरो अध्ययनको मुख्य उद्देश्य ART केन्द्रहरुमा HIV केयर डेलिभरीमा फार्मासिष्टको भूमिका पत्ता लगाउनु हो, एचआईभी देखभाल वितरणमा संलग्न फार्मेसी कार्यबलको उपस्थिति पत्ता लगाउने, HIV देखभालमा फार्मासिष्टको संलग्नतामा ज्ञात सहजकर्ता र अवरोधहरूको पहिचान गर्न। यस उद्देश्यका लागि, मैले अन्तर्वार्ता गाईड अनुसरण गर्दै केहि प्रश्नहरू सोध्नु पर्छ । उत्तरदाताहरु द्वारा प्रदान गरिएको सूचना अत्यन्त गोप्यता संग राखिनेछ र उनीहरुको पहिचान खुलाउने छैन । कुनै आर्थिक लेनदेन गरिने छैन र यस अध्ययनमा तपाईको सहभागिता स्वैच्छिक हुनेछ। यदि तपाईं यस अध्ययनमा भाग लिन चाहानुहुन्छ भने कृपया तलको सहमति फारममा हस्ताक्षर गर्नुहोस्। कृपया तलको कथन पढ्नुहोस्।

समय आवश्यक (अनुमानित): 30-60 **मिनेट**

वक्तव्य:

१. मैले बुझें कि मेरो अन्तर्वार्ता रेकर्ड हुन सक्छ।

२. म बुझ्दछु कि अध्ययनको क्रममा सकलन गरिएको डाटाले अन्य अन्वेषकहरू र नियामक प्राधिकरणहरूले हेरिरहेको हुन सक्छ।

३ म यो अन्तर्वार्ताको कारण बुझ्छु र यसमा भाग लिन इच्छुक र खुशी छु।

४ यदि म यो अन्तर्वार्तामा भाग लिन सहमत छु भने मँ बुझ्छु मलाई के गर्नु पर्ने हुन्छ।

५ मैले दिएको अन्तर्वार्ता र यसमा समावेश गरिएको जानकारी पूर्ण रूपमा परियोजना द्वारा परिभाषित उद्देश्यहरूको लागि प्रयोग गरिनेछ ।

६ मलाई थाहा छ मसँग कुनै पनि समयमा अन्तर्वार्ता छोड्ने वा कुनै प्रश्नको उत्तर दिन अस्वीकार गर्ने अधिकार छ।

७ यदि म यस अन्तर्वार्तामा भाग लिन सहमत भएन भने म बुझ्दछु कि अनुसन्धानकर्ताहरूले न त भविष्यमा कुनै पनि चिकित्सा सेवा कर्मीहरू द्वारा त्यसो गर्दा मलाई दण्ड दिइने छ।

Scanned with CamScanner

हम यस अन्तर्वार्तामा भाग लिन स्वेच्छाले सहमत छु।

[illegible] .... 2077-10-25 ....

हस्ताक्षर मिति

Scanned with CamScanner

सूचित सहमति फारम

नमस्ते, म आयुष्मा शाही, काठमाडौं विश्वविद्यालयको एम. फार्म विद्यार्थी हुँ। मेरो शैक्षिक पाठ्यक्रमको अंशको रूपमा, " PREDICTORS OF KNOWLEDGE, ATTITUDE, PRACTICE AND ADHERENCE OF ANTIRETROVIRAL THERAPY AND QUALITATIVE INSIGHT OF POTENTIAL ROLES OF PHARMACISTS IN HIV/AIDS CARE DELIVERY" शीर्षकको अनुसन्धान गर्दै छु। मेरो अध्ययनको मुख्य उद्देश्य ART केन्द्रहरुमा HIV केयर डेलिभरीमा फार्मासिष्टको भूमिका पत्ता लगाउनु हो, एचआईभी देखभाल वितरणमा संलग्न फार्मेसी कार्यबलको उपस्थिति पत्ता लगाउने, HIV देखभालमा फार्मासिष्टको संलग्नतामा ज्ञात सहजकर्ता र अवरोधहरूको पहिचान गर्न। यस उद्देश्यका लागि, मैले अन्तर्वार्ता गाईड अनुसरण गर्दै केहि प्रश्नहरू सोध्नु पर्छ । उत्तरदाताहरु द्वारा प्रदान गरिएको सूचना अत्यन्त गोप्यता संग राखिनेछ र उनीहरुको पहिचान खुलाउने छैन । कुनै आर्थिक लेनदेन गरिने छैन र यस अध्ययनमा तपाईको सहभागिता स्वैच्छिक हुनेछ। यदि तपाईं यस अध्ययनमा भाग लिन चाहानुहुन्छ भने कृपया तलको सहमति फारममा हस्ताक्षर गर्नुहोस्। कृपया तलको कथन पढ्नुहोस्।

समय आवश्यक (अनुमानित): 30-60 मिनेट

वक्तव्य:

१. मैले बुझें कि मेरो अन्तर्वार्ता रेकर्ड हुन सक्छ।

२. म बुझ्दछु कि अध्ययनको क्रममा सकलन गरिएको डाटाले अन्य अन्वेषकहरू र नियामक प्राधिकरणहरूले हेरिरहेको हुन सक्छ।

३ म यो अन्तर्वार्ताको कारण बुझ्छु र यसमा भाग लिन इच्छुक र खुशी छु।

४ यदि म यो अन्तर्वार्तामा भाग लिन सहमत छु भने मँ बुझ्छु मलाई के गर्नु पर्ने हुन्छ।

५ मैले दिएको अन्तर्वार्ता र यसमा समावेश गरिएको जानकारी पूर्ण रूपमा परियोजना द्वारा परिभाषित उद्देश्यहरूको लागि प्रयोग गरिनेछ ।

६ मलाई थाहा छ मसँग कुनै पनि समयमा अन्तर्वार्ता छोड्ने वा कुनै प्रश्नको उत्तर दिन अस्वीकार गर्ने अधिकार छ।

७ यदि म यस अन्तर्वार्तामा भाग लिन सहमत भएन भने म बुझ्दछु कि अनुसन्धानकर्ताहरूले न त भविष्यमा कुनै पनि चिकित्सा सेवा कर्मीहरू द्वारा त्यसो गर्दा मलाई दण्ड दिइने छ।

Scanned with CamScanner

म यस अन्तर्वार्तामा भाग लिन स्वेच्छाले सहमत छु।

[illegible]
066|60|26
....

हस्ताक्षर

....

मिति

Scanned with CamScanner

सूचित सहमति फारम

नमस्ते, म आयुष्मा शाही, काठमाडौं विश्वविद्यालयको एम. फार्म विद्यार्थी हुँ। मेरो शैक्षिक पाठ्यक्रमको अंशको रूपमा, " PREDICTORS OF KNOWLEDGE, ATTITUDE, PRACTICE AND ADHERENCE OF ANTIRETROVIRAL THERAPY AND QUALITATIVE INSIGHT OF POTENTIAL ROLES OF PHARMACISTS IN HIV/AIDS CARE DELIVERY" शीर्षकको अनुसन्धान गर्दै छु। मेरो अध्ययनको मुख्य उद्देश्य ART केन्द्रहरुमा HIV केयर डेलिभरीमा फार्मासिष्टको भूमिका पत्ता लगाउनु हो, एचआईभी देखभाल वितरणमा संलग्न फार्मेसी कार्यबलको उपस्थिति पत्ता लगाउने, HIV देखभालमा फार्मासिष्टको संलग्नतामा ज्ञात सहजकर्ता र अवरोधहरूको पहिचान गर्न। यस उद्देश्यका लागि, मैले अन्तर्वार्ता गाईड अनुसरण गर्दै केहि प्रश्नहरू सोध्नु पर्छ । उत्तरदाताहरु द्वारा प्रदान गरिएको सूचना अत्यन्त गोप्यता संग राखिनेछ र उनीहरुको पहिचान खुलाउने छैन । कुनै आर्थिक लेनदेन गरिने छैन र यस अध्ययनमा तपाईको सहभागिता स्वैच्छिक हुनेछ। यदि तपाईं यस अध्ययनमा भाग लिन चाहानुहुन्छ भने कृपया तलको सहमति फारममा हस्ताक्षर गर्नुहोस्। कृपया तलको कथन पढ्नुहोस्।

समय आवश्यक (अनुमानित): 30-60 मिनेट

वक्तव्य:

१. मैले बुझें कि मेरो अन्तर्वार्ता रेकर्ड हुन सक्छ।

२. म बुझ्दछु कि अध्ययनको क्रममा सकलन गरिएको डाटाले अन्य अन्वेषकहरू र नियामक प्राधिकरणहरूले हेरिरहेको हुन सक्छ।

३ म यो अन्तर्वार्ताको कारण बुझ्छु र यसमा भाग लिन इच्छुक र खुशी छु।

४ यदि म यो अन्तर्वार्तामा भाग लिन सहमत छु भने मैँ बुझ्छु मलाई के गर्नु पर्ने हुन्छ।

५ मैले दिएको अन्तर्वार्ता र यसमा समावेश गरिएको जानकारी पूर्ण रूपमा परियोजना द्वारा परिभाषित उद्देश्यहरूको लागि प्रयोग गरिनेछ ।

६ मलाई थाहा छ मसँग कुनै पनि समयमा अन्तर्वार्ता छोड्ने वा कुनै प्रश्नको उत्तर दिन अस्वीकार गर्ने अधिकार छ।

७ यदि म यस अन्तर्वार्तामा भाग लिन सहमत भएन भने म बुझ्दछु कि अनुसन्धानकर्ताहरूले न त भविष्यमा कुनै पनि चिकित्सा सेवा कर्मीहरू द्वारा त्यसो गर्दा मलाई दण्ड दिइने छ।

Scanned with CamScanner

म यस अन्तर्वार्तामा भाग लिन स्वेच्छाले सहमत छु।

.... २०८०.१०-२५.

हस्ताक्षर .... मिति
