# Supplementary material for: Potential roles of pharmacists in HIV/AIDS care delivery in Nepal: A qualitative study
Source: PLoS One. 2023 Jan 6;18(1):e0280160. doi: 10.1371/journal.pone.0280160 (PMC9821491; doi:10.1371/journal.pone.0280160)
Supplement: S2 File — Ethical approval from NHRC. (PDF) [file pone.0280160.s002.pdf]

Government of Nepal
Nepal Health Research Council (NHRC)
Estd. 1991

Ref. No.: 1891

Date: 20 January 2021

Dr. Sweta Shrestha

Principal Investigator

Kathmandu University

Ref: Approval of research proposal

Dear Dr. Shrestha,

This is to certify that the following protocol and related documents have been reviewed and granted approval by the Expedited Review Sub-Committee for implementation.

**ERB Protocol Registration No.**	834/2020 P	**Sponsor Protocol No**	NA
**Principal Investigator/s**	Dr. Sweta Shrestha	**Sponsor Institution**	University Grant Commission
**Title**	Knowledge, Attitude, Practice and Adherence of Antiretroviral Therapy and a Qualitative Insight of Potential Roles of Pharmacists in HIV/AIDS Care Delivery		
**Protocol Version No**	NA	**Version Date**	NA
**Other Documents**	1. Data collection tools<br>2. Acceptance letter from the study site<br>3. Donor agreement letter	**Risk Category**	Minimal risk
**Expedited Review**	Proposal √<br>Amendment ☐<br>Re-submitted ☐<br>**Meeting Date:** 18 January 2021	**Duration of Approval**<br>18 January 2021 to 18 January 2022	**Frequency of continuing review**<br>At least once in a year
**Total budget of research**	**NRs 3,00,000.00**		
**Ethical review processing fee**	**NRs 10,000.00**		

Investigator Responsibilities

- Any amendments shall be approved from the ERB before implementing them

Tel: +977 1 4254220, Fax: +977 1 4262469, Ramshah Path, PO Box: 7626, Kathmandu, Nepal
Website: http://www.nhrc.gov.np, E-mail: nhrc@nhrc.gov.np

Ref. No.: 1891

•	Submit progress report every 3 months
•	Submit final report after completion of protocol procedures at the study site
•	Report protocol deviation / violation within 7 days
•	Comply with all relevant international and NHRC guidelines
•	Abide by the principles of Good Clinical Practice and ethical conduct of the research

If you have any questions, please contact the Ethical Review M & E Section at NHRC.

Thanking you,

Dr. Pradip Gyanwali
Member-Secretary
(Executive Chief)
